# Growth of β-NaYF_4_:Eu^3+^ Crystals by the Solvothermal Method with the Aid of Oleic Acid and Their Photoluminescence Properties

**DOI:** 10.3390/ma12223711

**Published:** 2019-11-11

**Authors:** Jianhua Huang, Xiaojing Wang, An Shao, Guoping Du, Nan Chen

**Affiliations:** 1 School of Materials Science and Engineering, Nanchang University, Nanchang 330031, China; 2 Hunan Engineering Laboratory for Control and Optimization of PV Systems, Hunan Vocational Institute of Technology, Xiangtan 411104, China; 3 Queen Mary School, Nanchang University, Nanchang 330031, China

**Keywords:** lanthanide-doped fluoride, Eu^3+^-doped β-NaYF_4_, photoluminescence, solvothermal method

## Abstract

Lanthanide-doped hexagonal β-NaYF_4_ crystals have received much attention in recent years due to their excellent photoluminescence properties. However, lanthanide-doped β-NaYF_4_ crystals with micron and submicron scales as well as uniform morphology have received less attention. In this study, Eu^3+^-doped β-NaYF_4_ (β-NaYF_4_:Eu^3+^) crystals of micron and submicron size scales were synthesized using the solvothermal method with ethylene glycol as the solvent. The β-NaYF_4_:Eu^3+^ crystals were highly crystallized. A comparison of the characteristics of the β-NaYF_4_:Eu^3+^ crystals synthesized with and without the use of oleic acid as a surfactant was conducted. It was found that the utilization of oleic acid as a surfactant during their synthesis greatly decreased their particle size from micron to submicron scale, while adding a small amount of ethanol further reduced their particle size. In addition, they exhibited much smoother surfaces and more uniform morphologies, which were hexagonal prism bipyramids. The microstructural characteristics and photoluminescence properties of the β-NaYF_4_:Eu^3+^ crystals were studied in detail. Results showed that β-NaYF_4_:Eu^3+^ crystals prepared with the aid of oleic acid as a surfactant during their synthesis exhibited stronger photoluminescence.

## 1. Introduction

NaYF_4_ has two polymorphic forms: α- (cubic) and β-phase (hexagonal) structures. In recent years, NaYF_4_—especially the β phase—has been considered one of the most effective host materials for performing multicolor down-conversion (DC) and up-conversion (UC) photoluminescence of lanthanide ions (Ln^3+^) due to its unique properties [1], including low phonon energy (<400 cm^−1^) [2], high thermal and optical stability, good transparency, low non-radiative decay rate, and high radiative emission efficiency [3]. Ln^3+^-doped NaYF_4_ phosphors have been extensively studied [4,5,6,7,8,9,10] in both micro- and nano-sized scales because of their potential applications in light-emitting diodes (LEDs) [11], lasers [12], display devices [9], biological labels [13,14,15], solar cells [16,17], and many others.

The space groups of α- and β-NaYF_4_ are Fm3m (Z = 4) and P6 (Z = 1), respectively. The α-NaYF_4_ is isomorphic with CaF_2_ in which the sites of Ca^2+^ ions are substituted randomly by 1/2 Na^+^ ions and 1/2 Y^3+^ ions and the O_h_ symmetry of the cationic site has been retained [18]. Three types of cation sites exist in β-NaYF_4_: 1a sites are occupied by Y^3+^, 1f sites are occupied by 1/2Y^3+^ and 1/2Na^+^ ions randomly, while 2h sites are usually occupied by Na^+^ ions only [19,20]. The symmetry of the two Y^3+^ sites is C_3h_. Thus, the phase transition from α-NaYF_4_ to β-NaYF_4_ must overcome energy barriers of cation rearrangement, and it, is therefore a disorder-to-order arrangement process. It has been found that β-NaYF_4_ has a more stable crystal structure and better photoluminescence properties than α-NaYF_4_ [1,2,3], which has resulted in β-NaYF_4_ receiving more attention from researchers. However, Ln^3+^ ions (e.g., Eu^3+^, Tb^3+^, Dy^3+^, etc.) have a relatively small optical absorption cross section and a narrow absorption spectrum because of the forbidden nature of the f–f transition. Thus the intrinsic photoluminescence of Ln^3+^ ions in Ln^3+^-doped β-NaYF_4_ phosphors is limited [21]. So far, numerous methods have been studied to enhance the absorption and photoluminescence intensity of Ln^3+^-doped β-NaYF_4_ phosphors [22,23,24], including multiple types of Ln^3+^-doping and sensitization with organic ligands [8,25,26]. In this study, a surfactant was employed to control the morphology of the Ln^3+^-doped β-NaYF_4_ phosphors, and their photoluminescence properties were enhanced.

Ln^3+^-doped β-NaYF_4_ phosphors, in both micro-sized and nano-sized scales, have been previously synthesized using different methods, such as the molten salt method [27], thermal decomposition of organic precursors [28], sol–gel [17,29], and the hydrothermal/solvothermal method [30]. Some researchers [2,12,30,31] prepared NaYF_4_ crystals using the hydrothermal/solvothermal method in a water/alcohol/oleic acid system, while others [32] prepared two kinds of NaYF_4_ particles with different phase composition using an adjustable solvothermal method and by changing reaction temperature and molar ratio (Y^3+^/F^−^) with a different phase composition. NaYF_4_ nanorods, nanotubes, and nanodisks were prepared using oleic acid as hydrothermal synthesis-mediated agent [33], and UC β-NaYF_4_:Yb^3+^/Er^3+^ microcrystals were synthesized using the hydrothermal method at 220 °C [34]. However, it is still difficult to obtain Ln^3+^-doped β-NaYF_4_ particles with uniform and regular shape, high crystallinity, and strong photoluminescence performance. Moreover, these reported hydrothermal/solvothermal processes need either a high temperature or a long reaction time, which is neither economical nor environmentally friendly.

In this study, a simple and convenient solvothermal method was employed to synthesize highly dispersed Eu^3+^-doped β-NaYF_4_ crystals of micron and submicron size, with controlled morphology and strong photoluminescence. The preparation method was based on our previous work of α-NaYF_4_:Eu^3+^, which had used ethylene glycol/ethanol as a solvent [8]. Oleic acid (OA) was used as a surfactant to control the shape and morphology of the β-NaYF_4_:Eu^3+^ microcrystals with enhanced photoluminescence properties. Hexagonal prism bipyramidal β-NaYF_4_:Eu^3+^ crystals with uniform particle size of micron and submicron scale were successfully synthesized. The different characteristics of the phase structures, microstructural characteristics, and photoluminescence properties between the as-synthesized β-NaYF_4_:Eu^3+^ particles and those modified by OA were compared in detail. The mechanisms for their shape control, morphology optimization, and photoluminescence enhancement are discussed.

## 2. Method

### 2.1. Materials

Yttrium nitrate (Y(NO_3_)_3_·6H_2_O, 99.99%), europium nitrate (Eu(NO_3_)_3_·6H_2_O, 99.99%), sodium nitrate (NaNO_3_) (analytical grade, A.R.), and ammonium fluoride (NH_4_F) (A.R.) were used as the raw materials. OA (A.R.) was used as a surfactant, and ethylene glycol (EG, C_2_H_6_O_2_, A.R.) and absolute ethanol (C_2_H_5_OH, A.R.) were used as the solvents. All chemicals were used as received, without further purification. Deionized water was used throughout this work.

### 2.2. Synthesis of β-NaYF_4_:Eu^3+^ Crystals without Surfactant

The β-NaYF_4_:Eu^3+^ crystals were synthesized using a solvothermal method as previously described [8]. The doping concentration was 5 atm% with the formula β-NaY_0.95_Eu_0.05_F_4_. In a typical procedure, Y(NO_3_)_3_·6H_2_O (0.7277 g, or 1.9 mmol), Eu(NO_3_)_3_·6H_2_O (0.0446 g, or 0.1 mmol), and NaNO_3_ (0.1700 g, or 2 mmol) were mixed and thoroughly dissolved in 15 mL EG to form a clear solution A at room temperature. NH_4_F (1.7779 g, or 48 mmol) was added to a 15 mL EG solution to form solution B at room temperature. It was found that the phase structure of the synthesized NaYF_4_ strongly depended on the Y^3+^:F^−^ molar ratio in the reaction system. To obtain β-NaYF_4_:Eu^3+^ crystals, the molar ratio of Ln^3+^ ions (Y^3+^ + Eu^3+^) in solution A to F^−^ ions in solution B was set as Ln^3+^:F^−^ = 1:24. Solutions A and B were mixed under vigorous stirring for 2 h to form a mixture solution, which was poured into a 50 mL Teflon-lined stainless steel autoclave and heated at 180 °C for 6 h to synthesize β-NaYF_4_:Eu^3+^ crystals. After they were cooled down, the resultant β-NaYF_4_:Eu^3+^ crystals were collected in a similar manner as described previously [8]. The undoped β-NaYF_4_ crystals were synthesized as the standard sample, following the above procedure, except that no Eu^3+^ was used.

### 2.3. Synthesis of β-NaYF_4_:Eu^3+^ Crystals with the Aid of an OA Surfactant

OA was introduced into the synthesis as a surfactant to control the morphology and improve the photoluminescence of the β-NaYF_4_:Eu^3+^ crystals. The synthesis procedure was the same as the above procedure for non-surfactant synthesis (Section 2.2), with the following two modifications. First, 5 mL of OA was added to solution A. Second, the 15 mL of EG of solution A had two sets of OA additions: (1) 15 mL of EG, or (2) 2.5 mL of absolute ethanol and 12.5 mL of EG. Thus, two β-NaYF_4_:Eu^3+^ samples modified by an OA surfactant were synthesized with the two sets of OA additions.

### 2.4. Characterization

X-ray diffraction (XRD, PANalytical, Empyrean) and field emission scanning electron microscopy (FESEM, FEI-Quanta200F, Thermo Fisher, Waltham, MA, USA) were used to study the phase structures and observe the microstructural characteristics of the β-NaYF_4_:Eu^3+^ crystals. The X-ray diffractometer, equipped with graphite-monochromatized Cu Kα radiation (λ = 1.5406 Å) was operated at the scanning step of 0.0131303° in the 2θ range from 10° to 90°. The photoluminescence excitation and emission spectra were recorded with a Hitachi F-4600 fluorescence spectrophotometer (Hitachi, Tokyo, Japan) equipped with a xenon lamp source. All of the measurements in this work were conducted at room temperature.

## 3. Results and Discussion

### 3.1. Phase Structures and Microstructural Characteristics

**Figure 1:**
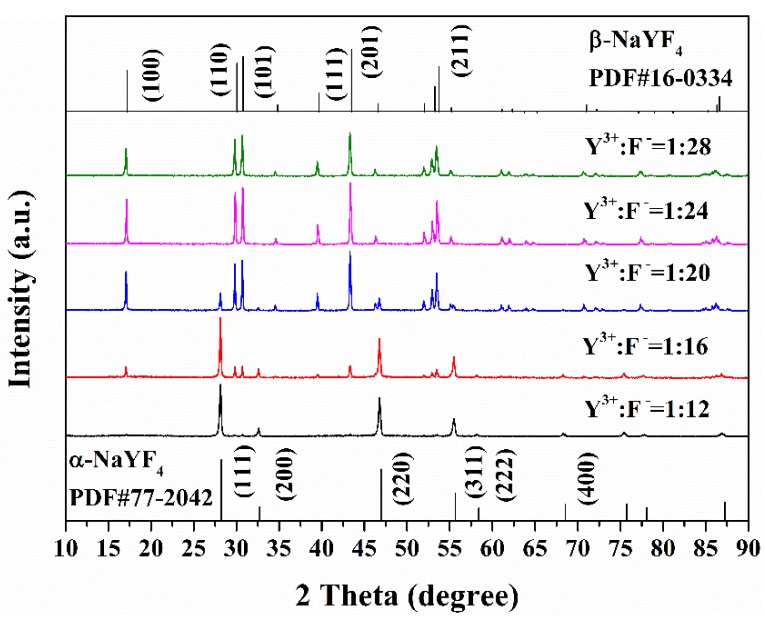
XRD patterns of NaYF_4_ prepared with different Y^3+^:F^−^ molar ratios in the reaction solution.

Figure 1 shows the XRD patterns of the as-synthesized NaYF_4_ crystals prepared using different Y^3+^:F^−^ molar ratios. The Joint Committee on Powder Diffraction Standards (JCPDS) data for the cubic (α-NaYF_4_, PDF#77-2042) and hexagonal (β-NaYF_4_, PDF#16-0334) crystal structure of NaYF_4_ are included in Figure 1 for reference. As shown in Figure 1, only the α-NaYF_4_ phase could be obtained when Y^3+^:F^−^ = 1:12, while mixed phases of α-NaYF_4_ and β-NaYF_4_ were present when Y^3+^:F^−^ = 1:16 and 1:20. When Y^3+^:F^−^ ≥ 1:24, only the β-NaYF_4_ phase was obtained. This suggests that the crystal structure of NaYF_4_ strongly depends on the Y^3+^:F^−^ molar ratios in the reaction solution. For this reason, the Y^3+^:F^−^ ratio was set at 1:24 to synthesize β-NaYF_4_:Eu^3+^ in this study.

**Figure 2:**
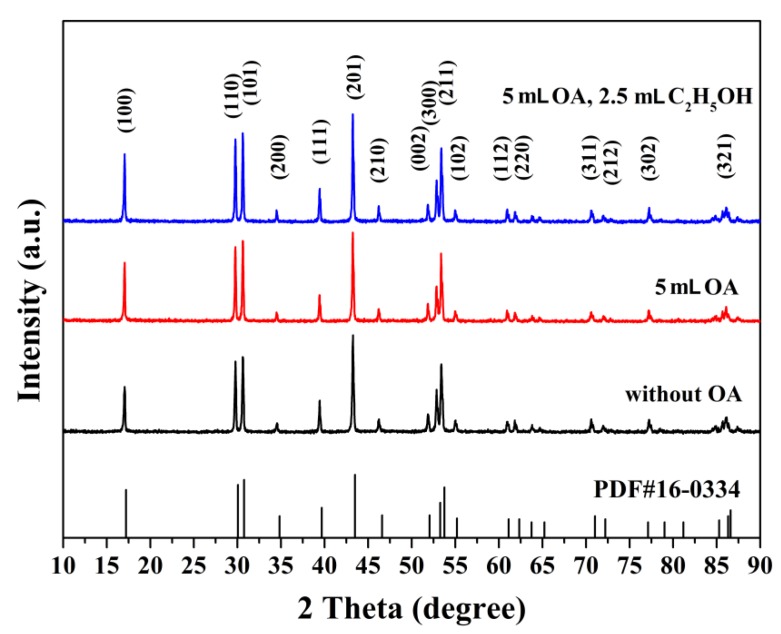
XRD patterns of β-NaYF_4_:Eu^3+^, prepared without oleic acid (OA), with 5 mL OA, and with 5 mL OA + 2.5 mL C_2_H_5_OH.

Figure 2 shows the XRD patterns of the as-synthesized β-NaYF_4_:Eu^3+^ crystals prepared without OA, with 5 mL OA and with 5 mL OA + 2.5 mL C_2_H_5_OH. All of the diffraction peaks (Figure 2) can be readily indexed to the pure hexagonal NaYF_4_ phase, and no secondary phase was observed in all samples. The sharp diffraction peaks in Figure 2 imply that all of the β-NaYF_4_:Eu^3+^ particles were well crystallized. As seen in Figure 2, all of the XRD patterns were relatively similar, which suggests that the utilization of an OA surfactant did not have a noticeable impact on the crystal structures of the β-NaYF_4_:Eu^3+^ crystals. Furthermore, the use of C_2_H_5_OH also had little influence on their crystal structures.

**Figure 3:**
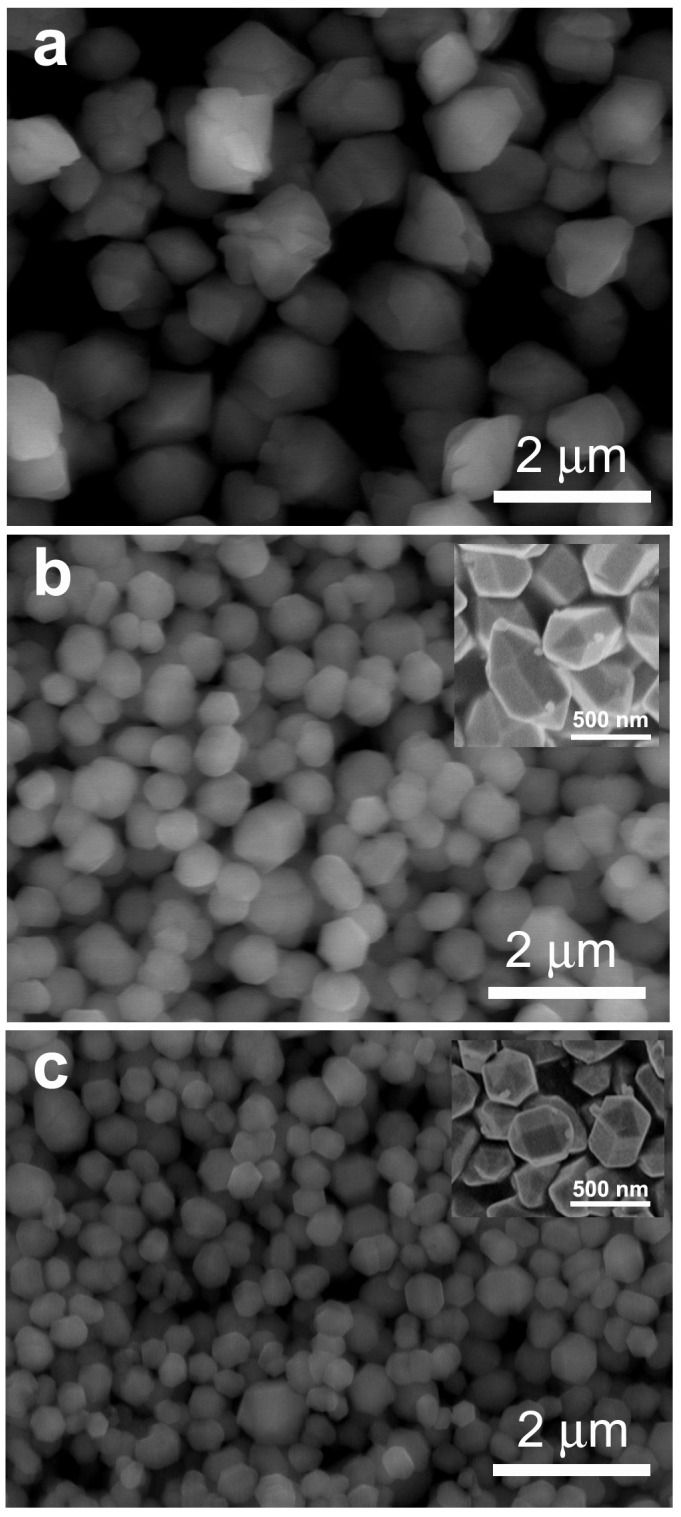
SEM images of β-NaYF_4_:Eu^3+^ prepared without OA (**a**), with 5 mL OA (**b**), and with 5 mL OA + 2.5 mL C_2_H_5_OH (**c**).

The SEM images in Figure 3 show the microstructural characteristics of the as-prepared β-NaYF_4_:Eu^3+^ particles. All of the β-NaYF_4_:Eu^3+^ particles were highly dispersible. The β-NaYF_4_:Eu^3+^ particles prepared without an OA surfactant were the largest (Figure 3a), which was about 1.5 μm. As shown in Figure 3b,c, the utilization of an OA surfactant in the reaction solution greatly reduced the size of the β-NaYF_4_:Eu^3+^ particles. The average NaYF_4_:Eu^3+^ particle size, prepared with 5 mL of OA and 5 mL of OA + 2.5 mL of C_2_H_5_OH was about 650 nm and 500 nm (Figure 3b,c), respectively. Thus, adding C_2_H_5_OH further reduced the size of the β-NaYF_4_:Eu^3+^ particles.

**Figure 4:**
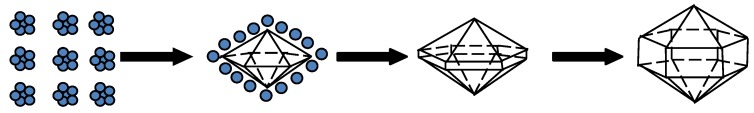
Illustration of the formation and evolution process of the β-NaYF_4_:Eu^3+^ hexagonal prism bipyramids (redrawn under the written permission of Prof. Chunhua Lu [34]).

As seen in Figure 3a, the β-NaYF_4_:Eu^3+^ particles, prepared without using an OA surfactant had irregular shapes with rough surfaces. However, the β-NaYF_4_:Eu^3+^ particles prepared with an OA surfactant were regular and uniform hexagonal prism bipyramids [34] with smooth surfaces (insets of Figure 3b,c). Thus, the OA surfactant not only reduced the particle size of β-NaYF_4_:Eu^3+^ from the micrometer range to submicron scale, it also greatly improved their morphologies. Note that the hexagonal prism bipyramid shape is derived from top and bottom halves, where each half is a six-sided prism. The hexagonal prism bipyramid structure of β-NaYF_4_:Eu^3+^ found in this experiment is similar to the β-NaYF_4_:Yb,Er micro-bipyramids shown by Ding et al. [34]. Their research [34] also proposed a possible morphology evolution mechanism for the β-NaYF_4_:Yb,Er micro-bipyramids, as illustrated in Figure 4. As pointed out by Liang et al. [2], the OA molecules are coated onto the outer face of the generated NaYF_4_ particles during the early reaction process through the interaction between Ln^3+^ (Y^3+^, Eu^3+^) and the carboxyl of the OA, with the hydrophobic alkyl chains left outside. This interaction can be weakened under high temperature and pressure during the solvothermal process to allow for the Ln^3+^ ions to be released gradually. The cubic α-NaYF_4_ is grown first, and these particles usually have an isotropic shape. Because α-NaYF_4_ is unstable, a more stable β-NaYF_4_ is formed through an α→β phase transformation during the ongoing reaction process [34]. Eventually, hexagonal prisms are formed [34].

The main reason for the reduced particle size of β-NaYF_4_:Eu^3+^ when using a surfactant is that OA covers the surface of the particles through the interaction between carboxyl and Eu^3+^ ions (Figure 3). Adding a small amount of C_2_H_5_OH into the reaction solution can further reduce the particle size because C_2_H_5_OH has a low boiling point, which boosts the internal pressure and consequentially improves the activity of ions. As a result, a large number of crystal nuclei form rapidly in a short time. Furthermore, the crystal nuclei will collide with each other violently, resulting in aggregation and uneven particle growth, which can be gradually uniformed to become microcrystals with increasing reaction time.

### 3.2. Photoluminescence Properties

**Figure 5:**
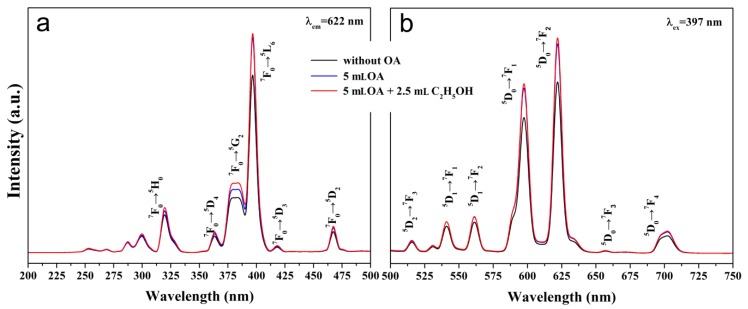
Excitation (**a**) and emission (**b**) spectra of the β-NaYF_4_:Eu^3+^ crystals prepared without OA, with 5 mL OA, and with 5 mL OA + 2.5 mL C_2_H_5_OH.

Figure 5 shows the photoluminescence excitation and emission spectra of the three β-NaYF_4_:Eu^3+^ samples prepared without OA, with 5 mL OA, and with 5 mL OA + 2.5 mL C_2_H_5_OH. All of the excitation peaks ranging from 200 to 500 nm are attributed to the characteristic excitation of Eu^3+^ ions at 320 nm (^7^F_0_→^5^H_0_), 364 nm (^7^F_0_→^5^D_4_), 379 nm (^7^F_0_→^5^G_2_), 397 nm (^7^F_0_→^5^L_6_), 418 nm (^7^F_0_→^5^D_3_), and 468 nm (^7^F_0_→^5^D_2_) [8]. The strongest excitation peak originates from the ^7^F_0_→^5^L_6_ transition. Similarly, the emission peaks excited by an exciting light at 397 nm are the characteristic emissions of Eu^3+^ ions, and they are assigned to the high-energy transition ^5^D_2_→^7^F_3_ at 515 nm and ^5^D_1_→^7^F_J_ (J = 0–2) at 530 nm, 541 nm, and 561 nm; low-energy transition ^5^D_0_→^7^F_J_ (J = 1–4) at 598 nm, 622 nm, 657 nm, and 702 nm, respectively [8,35]. The weaker emission peak at 598 nm corresponding to ^5^D_0_→^7^F_1_ is allowed by the magnetic dipole transitions due to Eu^3+^ ions, located at a site with inversion symmetry, while the stronger emission peak at 622 nm, corresponding to ^5^D_0_→^7^F_2_, is allowed by the electric dipole transitions that are a result of absence of inversion symmetry at the Eu^3+^ lattice site [36,37,38,39].

As seen in Figure 5, the utilization of OA during the synthesis enhanced the photoluminescence intensity of the β-NaYF_4_:Eu^3+^ crystals by about 20%. The β-NaYF_4_:Eu^3+^ crystals prepared with either 5 mL OA or with 5 mL OA + 2.5 mL C_2_H_5_OH had comparable photoluminescence intensity (Figure 5), with the latter being slightly higher than the former. The reason why OA can improve the photoluminescence of the β-NaYF_4_:Eu^3+^ crystals could be explained as follows. The β-NaYF_4_:Eu^3+^ crystals prepared with OA had a smoother surface and more integrated uniform shape than the ones prepared without using OA (Figure 3). Thus, the former should have a lower density of non-radiative traps on the surface than the latter, and therefore the former is expected to have stronger photoluminescence.

## 4. Conclusions

In summary, highly crystallized β-NaYF_4_:Eu^3+^ crystals with micron and submicron scale were successfully synthesized using the solvothermal method, with ethylene glycol as the solvent. The characteristics of the β-NaYF_4_:Eu^3+^ crystals synthesized with and without the use of oleic acid as a surfactant during their synthesis were compared. The utilization of OA as a surfactant during the synthesis of the β-NaYF_4_:Eu^3+^ crystals was found to greatly decrease their particle size from about 1.5 µm to about 650 nm. Adding small amount of ethanol could further decrease the particle to about 500 nm. In addition, the β-NaYF_4_:Eu^3+^ crystals synthesized with the aid of OA as a surfactant also exhibited much smoother surfaces and more uniform morphologies, which were hexagonal prism bipyramids. The present study also found that the β-NaYF_4_:Eu^3+^ crystals prepared with the aid of OA as a surfactant during their synthesis exhibited about 20% stronger photoluminescence than those synthesized without using an OA surfactant.
